# Mortality Risk Factors of Severely Injured Polytrauma Patients (Prehospital Mortality Prediction Score)

**DOI:** 10.3390/jcm12144724

**Published:** 2023-07-17

**Authors:** Jana Vorbeck, Manuel Bachmann, Helena Düsing, René Hartensuer

**Affiliations:** 1 Surgical Clinic II, Aschaffenburg-Alzenau Hospital, 63739 Aschaffenburg, Germanyrene.hartensuer@klinikum-ab-alz.de (R.H.); 2 Department of Trauma, Hand, and Reconstructive Surgery, University Hospital Münster, 48149 Münster, Germany

**Keywords:** polytrauma, risk factor, mortality risk score, trauma suite, emergency

## Abstract

The aim of this study was to analyze the mortality of polytrauma patients and identify prediction parameters. A further aim was to create from the results a score for the prehospital predictive evaluation of 30-day survival. The study was conducted with a retrospective, observational design and was carried out unicentrically at a Level 1 Trauma Center. During the 4-year investigation period, patients with an Injury Severity Score (ISS) ≥ 16 were examined and their demographic basic data, laboratory values, and vital parameters were recorded. The mortality data analysis was performed using Kaplan–Meier Analysis and Log-Rank tests. Cox regressions were carried out to determine influencing factors and Receiver Operating Characteristic (ROC) curves were plotted to establish limit values for potential influencing factors. All statistical tests were conducted at a significance level of *p* ≤ 0.05. Coronary Heart Disease (CHD), cardiopulmonary resuscitation (CPR), age at admission, sex, and Glasgow Coma Scale (GCS) had a significant impact on the survival of polytrauma patients. The identified prediction parameters were combined with the shock index (SI). The generated score showed a sensitivity of 93.1% and a specificity of 73.3% in predicting the mortality risk. The study was able to identify significant influencing prehospital risk factors on 30-day survival after polytrauma. A score created from these parameters showed higher specificity and sensitivity than other prediction scores. Further studies with a larger number of participants and the inclusion of slightly injured patients could verify these findings.

## 1. Introduction

It is widely known that polytrauma care is still associated with a high mortality risk. Standardized concepts for treatment in the trauma suite were introduced to improve the procedures [1]. The search for independent factors influencing mortality and the formation of prediction scores is the subject of current discussion. In addition to demographic basic data, the influences of previous illnesses, vital parameters, and laboratory test scores have been analyzed with sometimes contradictory results. In particular, various studies describe lactate as an influencing factor for survival after polytrauma [2,3,4,5], while others exclude its influence [6,7]. However, there is broad consensus in the literature that higher age and lower GCS predict an increase in mortality after polytrauma [8,9]. In addition, biological sex and blood pressure may also play an independent role [10,11,12]. There are various scores for predicting mortality risk, which, to a large extent, include the injury pattern, which is not yet fully known at the start of the treatment in the emergency room. The ISS solely describes the pattern of injury and does not consider vital parameters [13]. A complete and accurate assessment is only possible with imaging studies. The Revised Trauma Score (RTS) includes only three parameters, all of which are available prehospitally [14]. It is used in conjunction with the ISS to create the Trauma Score and Injury Severity Score (TRISS) [15]. However, TRISS cannot be used for a complete prehospital prediction due to the lack of imaging studies. The Rapid Emergency Medicine Score (REMS) only includes prehospital parameters but has a paradoxical relationship with mortality (a lower score is associated with apparently higher mortality) [16]. Additionally, there are various scores that only consider the presence of shock (SI, Reverse Shock Index, ACS/ATLS Hemorrhage Score). The latter cannot be fully assessed in a prehospital setting as an evaluation of urine output is necessary. Some other scores require various laboratory parameters for assessment, rendering them unsuitable for initial assessment (Acute Physiology And Chronic Health Evaluation Score II (APACHE II) [17], Revised Injury Severity Classification (RISC II) [18], Trauma Early Mortality Prediction Tool (TEMPT) [19]). A notable disparity can be observed between complex and clinically derived scores and previous simple prehospital prediction models without substantial statistical certainty.

The aim of the present study was to investigate independent factors influencing mortality within the first 30 days after injury in patients with an ISS > 16 and to create a score for predicting the probability of survival from the potential factors, which can provide information about the mortality risk. The performance of the score and its predictive ability was then compared with other established scores. We postulate that there are specific factors with a significant influence on the outcome and survival of patients after polytrauma, independent of the injury pattern. Furthermore, we assume that these predictors can be used to create a score with sensitive and specific information about mortality after polytrauma. Moreover, we expect that the developed score exhibits at least an equally good performance as the scores cited above. Additionally, we posit that it is possible to significantly estimate the mortality risk solely based on prehospital predictors.

## 2. Materials and Methods

The study was by design clinical, unicentric, retrospective, and observational. Only those patients who underwent trauma room treatment due to an accident during the recruitment period from 1 January 2018 to 31 December 2021 were recorded. Data collection took place in 2022 and 2023. In addition to the basic demographic data, such as age at the time of the accident and sex, height, and weight (to calculate the BMI), the profile of co-morbidities, and data on the stay (trauma suite, general care unit, intensive care unit, intermediate care unit) were also recorded as well as the circumstances and dates of death. Laboratory data and Blood Gas Analysis (BGA) data from the first blood sample taken in the trauma suite and primarily measured vital parameters were included in the calculations. Whether CPR took place after the accident and during the stay was also recorded.

Statistical calculations were performed using SPSS Statistics^®^ 22 for Windows [20] for statistical and graphical analysis. The test for statistical significance in the case of unequal distributions of nominally scaled data was carried out using the chi-square test and Fisher’s exact test, in the case of ordinally scaled data using the Mann–Whitney U-test, and in the case of normally distributed data using the t-test. *p*-values of ≤0.05 were assumed to be statistically significant. All tests were two-tailed. Kaplan–Meier curves for survival time analysis were generated for the analysis of overall survival and reintervention-free survival and a comparison was made using a log-rank test. Cox regression (layerwise comparison of accident mechanism methods, probability of stepwise inclusion 0.05–0.1, backward conditional) was used to determine predictors of survival. ROC curves were used to identify the thresholds for individual parameters when creating the score.

## 3. Results

**Figure 1:**
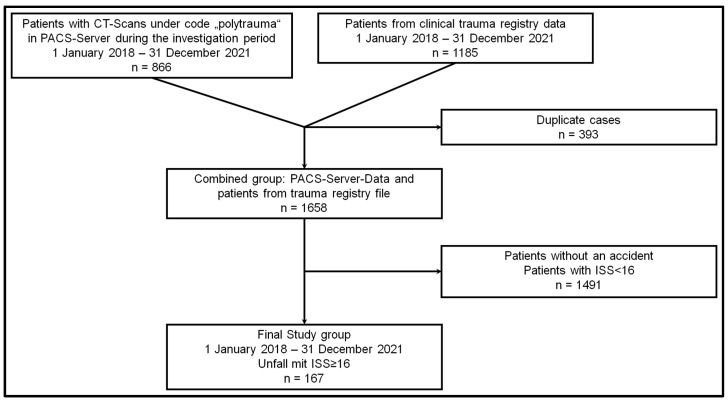
Flow Chart of the patient inclusion process: Out of 2051 cases initially considered, ultimately, 167 were included in the study. CT, Computed Tomography.

After excluding duplicate and miscoded cases and patients with an ISS below 16, the final study cohort of 167 patients was created from the hospital’s internal trauma registry database and data from the Picture Archiving and Communication System (PACS) server (Figure 1).

A total of 124 (74.3%) male and 43 (25.7%) female patients were enrolled. The average age of the patients was 56 years old (SD 21 years) and the average GCS was 11 (SD 5) (Table 1). 

The overview of co-morbidities/pre-existing conditions showed a large proportion of patients with arterial hypertension (42.5%) and hyperlipidemia (46.1%) (Table 2). 

A total of 134 patients survived and 33 died, with 30 of the deaths occurring within the first 30 days after the accident. The follow-up period was on average 289 days (median 75 days) for survivors and on average 3.63 days (median 0 days) for those who died within 30 days after the accident. 

**Figure 2:**
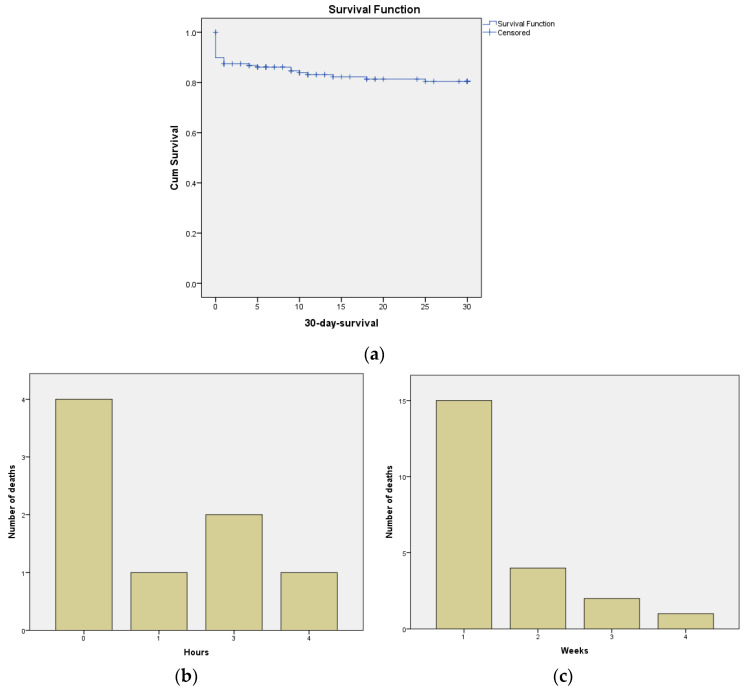
Representation of Mortality Rate within the First 30 Days: (**a**) Kaplan–Meier Curve of 30-day survival (Censored, refers to the status of patients for whom the event of interest (death) has not occurred within their personal follow-up duration.), (**b**) mortality rate in the first hours, and (**c**) mortality rate in the first weeks.

Panel (a) of Figure 2 demonstrates a significant increase in mortality during the initial days, as well as the subsequent mortality trend. In panels (b) and (c), the number of deaths within the first hours and the first weeks is depicted. The highest mortality rates are observed within the first hour and after 5 h in the first week.

To analyze potential influencing factors on mortality within 30 days after polytrauma, various parameters described above, especially those that have been described as influential in the literature, were included in a Cox regression. The Cox regression was performed using a backward conditional approach (stepwise elimination of the variable with the lowest influence/highest significance value). Changes in the significance of the same variable can be explained by interactions between variables, which can vary during the elimination steps. In the final step of this regression analysis, significant influences on 30-day survival were observed for patient age (*p* = 0.029), sex (*p* = 0.013), CHD (*p* = 0.015), CPR (*p* < 0.001), and admission GCS (*p* < 0.001) (Table 3).

**Figure 3:**
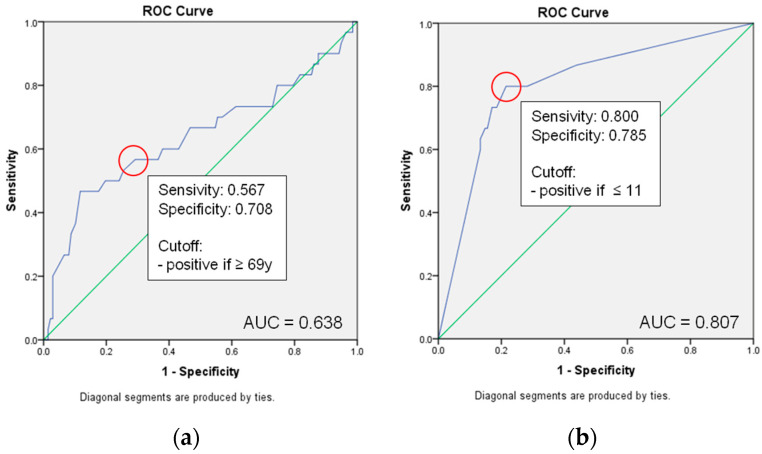
ROC Curves and Cutoff Values for 30-day survival: (**a**) age and (**b**) GCS.; green line, reference line.

The cutoff values of the influential metric parameters were determined by the ROC curves for age (cutoff: age 69 years and older) and GCS (cutoff: GCS 11 and below) shown in Figure 3.

**Figure 4:**
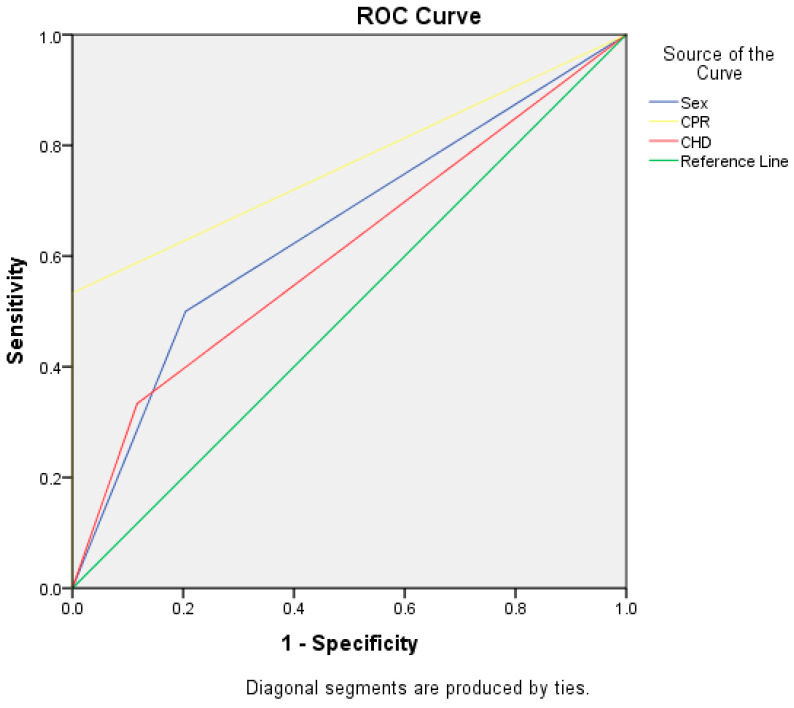
ROC Curves for 30-day survival: CPR, CHD, and Sex.

To complete the analysis, ROC curves for CPR (AUC = 0.767), CHD (AUC = 0.608), and Sex (AUC = 0.648) are given in Figure 4.

**Figure 5:**
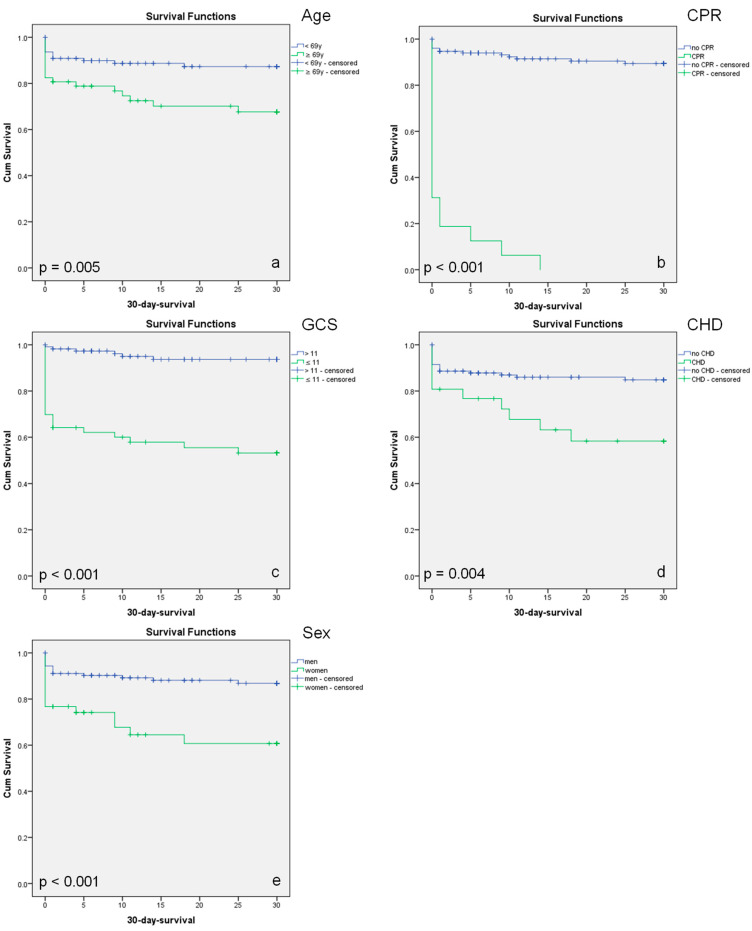
Kaplan–Meier Curves of Predictors: (**a**) Age in years, (**b**) CPR, (**c**) GCS, (**d**) CHD, and (**e**) Sex; Censored, refers to the status of patients for whom the event of interest (death) has not occurred within their personal follow-up duration.

To confirm the calculated thresholds and assess the significance of the nominal parameters, Kaplan–Meier analyses were conducted. Significant differences in 30-day survival were confirmed for age ≥ 69 years, CPR, GCS ≤ 11, existing CHD, and female sex (Figure 5).

In addition, the SI was determined to specifically include hemorrhagic emergencies. Subsequently, the total score was computed by assigning one point to each parameter described above if it was positive (existing CHD, CPR, age ≥ 69, GCS ≤ 11, female sex, SI ≥ 1). All the mentioned parameters can be assessed prehospitally, making this score a prehospital mortality prediction score (PMPS).

**Figure 6:**
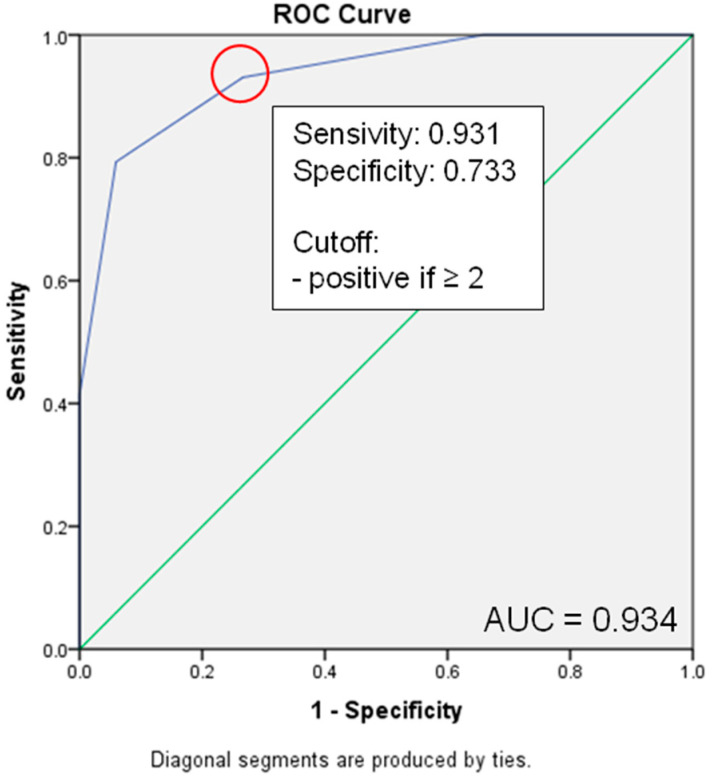
ROC Curve Score and Cutoff Value for 30-day survival. Green line, reference line.

After calculating the ROC curve, a significant increase in mortality could be predicted with a threshold of ≥2, achieving a sensitivity of 93.1% and a specificity of 73.3% (Figure 6). 

The PMPS calculation is illustrated in Table 4.

When the corresponding points are summed up, a total of 2 or more indicates a significantly increased risk of mortality.

**Figure 7:**
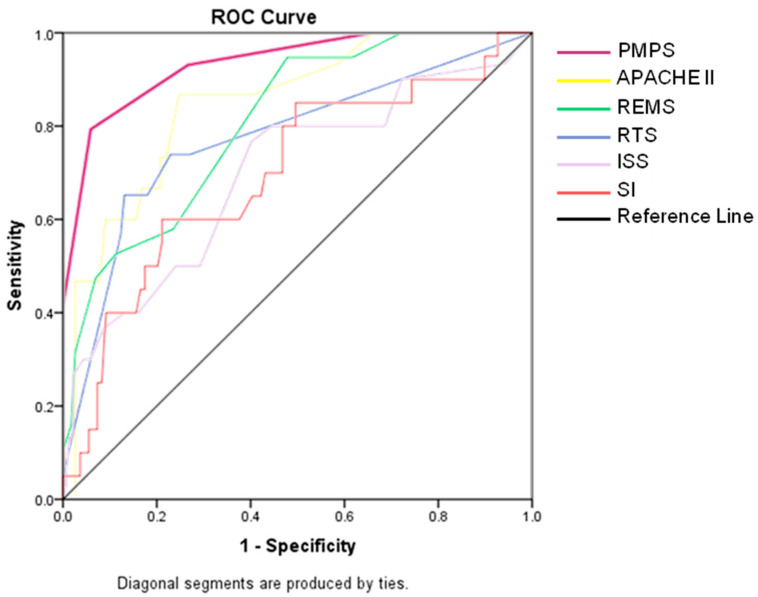
Comparison of different scores.

After adding up all the points, a minimum of 0 and a maximum of 6 points can be achieved. A higher score is associated with higher mortality. The threshold for a significant increase in mortality is ≥2 points. The AUC of the developed score (PMPS 0.934) was compared to both prehospital scores (SI (0.697), RTS (0.774) and clinical scores (APACHE II (0.846), REMS (0.808), ISS (0.697)) derived from the study cohort data, revealing a significantly better performance in mortality prediction (Figure 7).

In summary, a score with sensitive and specific significance for mortality within 30 days after polytrauma could be created from the significant influencing factors of CHD, resuscitation, Age, GCS, and sex in addition to the SI.

## 4. Discussion

### 4.1. Key Results

Our study was able to confirm the above-described risk factors of age, GCS, and biological sex and to define respective limit values for increased mortality. In addition, further factors influencing the 30-day survival were found. The presence of CHD and whether CPR had been carried out were also prognostic factors for increased mortality. A score with higher sensitivity and specificity and easier accessibility than comparable scores was created from the parameters mentioned and the SI. All the mentioned factors can be evaluated prehospitally, providing an early indication of the survival probability of polytrauma patients.

### 4.2. Limitations

The patients included in the study had already undergone trauma suite care before the start of the data collection. The determination of the severity of the injury and classification according to the ISS was carried out independently of the treatment plan and before the actual results were evaluated. A targeted selection of patients within the subgroup of the severely injured was therefore not carried out.

Only those with an ISS of 16 or more (the definition of polytrauma) [21,22] were included. However, this standardization should also serve to enable comparability with other studies and is therefore more useful than restrictive. In addition, only four patients under the age of 18 were included. Also, significantly fewer patients were included retrospectively than in large registry studies. The measured average serum lactate levels upon admission appear relatively low compared to other studies [4,5]. Therefore, any unmeasured influence on mortality should be considered with caution. The relatively small number of patients can influence the significance levels of variables in Cox regression due to random fluctuations in the data. To support the influence of variables, additional statistical techniques were employed. Assessing potential underlying CHD in severely injured, unconscious patients can be challenging in the prehospital setting. The reliable confirmation of CHD may be limited during this phase, even after gathering information from third-party sources, such as obtaining a medical history, reviewing medication plans, or checking for anticoagulation or stent identification.

### 4.3. Interpretation

The primary study objectives were achieved as both independent prehospital influencing factors were identified and a score was created from these factors. Various predictors from previous studies could also be confirmed in this study despite a smaller number of patients. Since, in principle, only events with a higher frequency were examined, the lower number of cases should not be considered a direct limitation. The non-standardized long-term follow-up period was addressed with a focus on 30-day survival.

Only two endpoints were examined to avoid an increase in the risk of false positive values [23]. Multiple testing was prevented by the linear and standardized approach of the analysis [24]. Values that were excluded in the previous examination level or testing were no longer relevant for the subsequent examinations. (Linear procedure: 1. Cox regression with all factors → 2. ROC curve for cutoff values of metric parameters → 3. Kaplan–Meier curve and score formation).

Based on this initial study, the applicability of the score appears feasible due to its simple composition from parameters that are already known prehospitally. The transferability to the group of all patients treated in the trauma suite must now be checked. It has already been proven that the ISS alone is not sufficient to fully carry out a patient assessment [25]. However, the composition of predictive factors can be partly proven by previous studies. The time of admission to the trauma suite (shift) does not influence survival (Table 3) [26]. The higher mortality of older people [27] and the lack of influence of BMI on the outcome [28] were also confirmed. In addition, the influence of lactate could not be verified (Table 3).

The present study was able to largely confirm previous statements in the literature and propose a score that can already make predictions prehospitally of mortality with high sensitivity and specificity, regardless of the injury pattern. 

The comparison with previous scores clearly demonstrated both the increased predictive power (Area Under Curve (AUC)) compared to solely prehospital assessable scores and the easier usability compared to well-performing ex post scores. The calculation of the RTS requires an additional complex mathematical formula despite having simple parameters [14], while the SI alone only describes a shock event and does not provide meaningful information for all polytrauma patients. ISS, RISC II, APACHE II, TRISS, REMS, ACS/ATLS, and TEMPT all exhibit good performance, but they cannot be assessed prehospitally. Thus, the PMPS fills this gap and can be utilized, for instance, for appropriate allocation to the corresponding level of care (from the prehospital setting to the appropriate hospital).

### 4.4. Generalizability

The results of the study can be projected to the general population to a limited extent. Due to the preselection according to the ISS score, the data can represent this subgroup of the severely injured. Due to the admissions procedure in the facility used in the present study, some injured children may have evaded the study (the children’s hospital has a separate emergency room). However, it seems safe to apply the findings to adults. When comparing the study data with the information from the TraumaRegister DGU^®^ for the corresponding years, it is noticeable that the patients in the present study were on average slightly older and the proportion of men was slightly higher [29]. However, limitations to the generalizability due to these small deviations cannot be determined.

## 5. Conclusions

The present study suggests that age (≥69 years), CPR, GCS (≤11), presence of CHD, and female sex significantly influence 30-day survival after polytrauma in adults. It was also shown that a sensitive and specific score (PMPS) for predicting mortality could be formed from these factors and the SI. We postulate that this score bridges the gap between simple prehospital and complex intrahospital trauma scores.

Further multicentre, prospective studies or the use of registry data (e.g., The German Trauma Registry) should be able to verify the applicability of this score and the individual determinants (weighing might be necessary), especially in patients with minor injuries and children, and could provide evidence for utilizing the score to simplify the decision-making process in the prehospital emergency setting for assigning patients to appropriate levels of care.

## Figures and Tables

**Table 1:** Patient Data.

Variable	Mean	Median	Maximum	Minimum	Valid *n*	Missing	Standard Deviation
age (years)	56	60	95	0	167	0	21
BMI (kg/m^2^)	26.32	25.38	54.08	17.71	106	61	4.79
height (cm)	174	175	200	83	107	60	14
weight (kg)	80	80	160	12	109	58	20
RR systolic (mmHG)	136	136	230	30	160	7	32
RR diastolic (mmHG)	81	80	160	10	150	17	19
temperature (°C)	36.2	36.4	38.5	34	117	50	0.8
breathing rate (1/min)	17	17	34	3	149	18	5
oxygen saturation (%)	96.49	98	100	20	160	7	7.2
heart rate (1/min)	88	83	170	50	159	8	22
GCS	11	14	15	3	165	2	5
NRS	5	5	10	0	53	114	2
pH	7.31	7.33	7.52	6.8	148	19	0.119
base excess	−2.14	0.7	8.2	−27.8	133	34	6.04
lactate (mmol/L)	2.7	2	14.7	0.5	143	24	2.39
CRP (mg/L)	4.56	1.4	85.1	0.6	165	2	11.68
troponin (pg/mL)	30.96	8	565	0	144	23	83.15
leukocytes (10^3^/µL)	11.68	10.57	41.16	3.27	166	1	5.82
erythrocytes (10^6^/µL)	4.58	4.51	16	1.6	166	1	1.11
hemoglobin (g/dL)	14.61	14	134	1.4	166	1	9.92
sodium (mmol/L)	139.68	140	149	120	165	2	3.35
potassium (mmol/L)	4.02	4	5.7	3	161	6	0.56
creatinine (mg/dL)	1.43	1,03	51	0.26	165	2	3.97
creatine kinase (U/L)	326.8	232	2807	62	159	8	355.81

BMI, Body Mass Index; RR, Blood Pressure; NRS, Numeric Rating Scale for Pain; CRP, C-Reactive Protein.

**Table 2:** Co-morbidities/Pre-existing conditions.

Patient Characteristic		Adjusted Count	Layer *n*%
hypertension	no hypertension	96	57.50%
	hypertension	71	42.50%
diabetes	no diabetes	144	86.20%
	diabetes type II	23	13.80%
COPD	no COPD	161	96.40%
	COPD	6	3.60%
hyperlipidemia	no hyperlipidemia	90	53.90%
	hyperlipidemia	77	46.10%
CHD	no CHD	141	84.40%
	CHD	26	15.60%
renal insufficiency	no renal insufficiency	153	91.60%
	renal insufficiency	14	8.40%
anticoagulation	no anticoagulation	129	77.20%
	Enoxaparin sodium	2	1.20%
	n.f.d.	5	3.00%
	ASA	17	10.20%
	*Phenprocoumon*	6	3.60%
	Clopidogrel	2	1.20%
	Apixaban	3	1.80%
	Rivaroxaban	2	1.20%
	Edoxaban	1	0.60%

COPD, Chronic Obstructive Pulmonary Disease; n.f.d., not further described; ASA, Acetylsalicylic Acid.

**Table 3:** Cox Regression 30-day survival.

Calculation Steps	Variables	Significance
Step 1	Age	0.073
	Sex	0.19
	pH	0.5
	Lactate	0.925
	Hemoglobin	0.58
	BE	0.463
	GFR	0.879
	Hypertension	0.32
	CHD	0.129
	COPD	0.366
	Diabetes	0.842
	Accident Mechanism	0.414
	Service Shift	0.99
	CPR	0.021
	GCS	0.033
Step 11	Age	0.029
	Sex	0.013
	CHD	0.015
	CPR	0
	GCS	0

BE, Base Excess; GFR, Glomerular Filtration Rate; Blood Pressure; COPD, Chronic Obstructive Pulmonary Disease.

**Table 4:** PMPS—Mortality Risk Assessment.

Condition/Risk Factor	Points
Coronary Heart Disease	1
Cardiopulmonary Resuscitation	1
Age ≥ 69 years	1
Glascow Coma Scale ≤ 11	1
Sex category (female)	1
Shock Index ≥ 1	1

## Data Availability

The data presented in this study are available on request from the corresponding author. The data are not publicly available due to restrictions imposed by the Bavarian Hospital Act and the responsible ethics committee of the Bavarian State Medical Association.
